# Dendritic Cells Activate and Mature after Infection with *Mycobacterium tuberculosis*

**DOI:** 10.1186/1756-0500-4-247

**Published:** 2011-07-21

**Authors:** Adane Mihret, Gezahagne Mamo, Mesfin Tafesse, Asrat Hailu, Shreemanta Parida

**Affiliations:** 1 Armauer Hansen Research Institute (AHRI), Addis Ababa, Ethiopia; 2 Faculty of Medicine, Addis Ababa University, Addis Ababa, Ethiopia; 3 Faculty of Veterinary Medicine, Addis Ababa, University, Addis Ababa, Ethiopia; 4 Max Planck Institute for Infection Biology, Berlin, Germany

**Keywords:** Dendritic cells, Mycobacterium tuberculosis, T cells, Activation, Flowctometry, CFSE, Proliferation

## Abstract

**Background:**

Dendritic cells (DCs) can take up an array of different antigens, including microorganisms which they can process and present more effectively than any other antigen presenting cell. However, whether the interaction between the human DC and *Mycobacterium tuberculosis *represents a defense mechanism by the invaded host, or helping the invader to evade the defense mechanism of the host is still not clearly understood.

**Findings:**

To analyze the interactions between *M. tuberculosis *and immune cells, human peripheral blood monocyte-derived immature DCs were infected with *M. tuberculosis *H37Rv wild type strain and flow cytometry was used to analyse cell surface expression markers. The ability of the *M. tuberculosis *infected DC to induce T cell proliferation using 5 and 6-carboxyfluorescein diacetate succinimidyl ester (CFSE) dilution technique was also investigated. DCs were found to internalize the mycobacteria and show dose dependent infection and necrosis with different multiplicity of infection. Flow cytometry analysis of cell surface expression markers CD40, CD54, CD80, CD83, CD86 and HLA DR in infected DC revealed significant (p < 0.05) up regulation following infection with *M. tuberculosis *in comparison to immature DC with no stimulation. Lipopolysaccharide (LPS) from *Salmonella abortus equi*, a known DC maturation agent, was used as a positive control and showed a comparable up regulation of cell surface markers as observed with *M. tuberculosis *infected DC. It was revealed that the *M. tuberculosis *infected DC induced T cell proliferation.

**Conclusion:**

These data clearly demonstrate that *M. tuberculosis *induces activation and maturation of human monocyte-derived immature DC as well as induces T cell proliferation *in vitro*.

## Background

*Mycobacterium tuberculosis *is one of the most ubiquitous and extraordinarily effective human pathogens, with one-third of the global population being infected. Worldwide, an estimated 8.8 million new cases of tuberculosis occurred annually, of which 3.9 million are smear positive [1]. Although effective therapy is now available through the WHO DOTS (Directly Observed Therapy, short course) program and the majority of the world's population is BCG vaccinated, the incidence of TB is still staggering. Clearly, a more effective vaccine against this disease is needed. A greater understanding of the immune response to the organism is necessary in order to develop a vaccine that will confer complete protection from infection and, hopefully, from reactivation of a pre-existing infection. In particular, the role of Dendritic cells (DCs) in the initiation of specific T cell immunity to *M. tuberculosis *has not been clearly elucidated.

DCs comprise a family of antigen presenting cells that act like 'conductors' of the immune response in their capacity to orchestrate signals derived from the different parts of the immune system [2]. DCs show a unique functional duality during their development, designed to ultimately provide secondary lymphoid tissues with useful information about the antigenic composition in the periphery. At the immature stages of development, DCs resident in peripheral tissues are specialized in antigen capture, acting as sentinel cells (high intracellular MHC II, endocytosis, phagocytosis, high CCR1, CCR5, and CCR6; low CCR7 and low CD40, CD54, CD80, CD83, CD86 and CD58).

After antigen uptake, DC rapidly migrate to the draining secondary lymphoid organ. During this migration, DC undergo a maturation process which is characterized by down-regulation of the capacity to capture antigen and up regulation of antigen processing and presentation, expression of costimulatory molecules and of dendritic morphology (high surface MHC II, low endocytosis, low phagocytosis, low CCR1, CCR5, CCR6, high CCR7 and high CD40, CD54, CD80, CD83, CD86 and CD58) [3].

Whether the encounter between the human dendritic cells and *M. tuberculosis *represents a defence mechanism by the invaded host, or helping the invader to evade the defence mechanism of the host is still not clearly understood. Henderson *et al *reported that human monocyte derived DC phagocytose *M. tuberculosis *efficiently, suggesting a role for this important cell in the early response to TB infection. Infection with this pathogen resulted in up regulation of MHC I and MHC II, CD40, CD54, CD58, and CD80 [4], a phenotype consistent with the activation of the DC, suggesting that infected DC produce cytokines that lead to maturation, and possibly to migration and antigen processing and presentation. In contrast to these findings, other workers have reported that *M. tuberculosis *inhibits maturation of human monocyte-derived dendritic cells *in vitro *and infected DCs show some up regulation of human monocyte-derived DC surface expression of maturation markers such as CD25, CD83, CD 40, CD80, CD86 and the antigen presenting molecules MHC I and MHC II. This was minimal compared with that induced by the maturation cocktail of TNFα, IL-1β, and PGE_2_. *M. tuberculosis *infected human monocyte-derived DC are compromised in their ability to activate naive T cells and were poor inducers of autologous T cell proliferation and cytokine production [5]. However, in this study our data demonstrate that immature human DC can take up *M. tuberculosis *bacilli *in vitro *and the infection induces DC activation, as measured by up regulation of cell surface markers accompanied by T cell proliferation.

## Materials and methods

### Media and Reagents

The medium used throughout the study was RPMI 1640 with L-glutamine (Sigma), supplemented with 1% penicillin streptomycin (Difco) and 10% FCS (Sigma), (RPMI-FCS). Human rGM-CSF and rIL 4 were purchased from Cytolab. LPS from *Salmonella abortus equi *was purchased from Sigma, and CFSE was purchased from Molecular Probes.

Monoclonal antibodies conjugated with fluorochromes against CD40 FITC (5C3 clone and mouse IgG_1, k _antibody composition), CD54 PE (HA58 clone and mouse IgG_1, k _antibody composition), CD80 PE (L307.4 clone and mouse IgG_1, k _antibody composition), CD83 FITC (HB15e clone and Mouse IgG_1, k _antibody composition), CD86 PE (FUN-1 clone and mouse IgG_1, k _antibody composition), HLA DR FITC (G46-6 clone and mouse IgG_2a, k _antibody composition), CD14 FITC (M5E_2 _clone and mouse IgG_2a,k _antibody composition), CD3 PE (UCHT1 clone and mouse IgG_1, k _antibody composition), FITC conjugated mouse IgG1 Isotype control (MOPC-21 clone and mouse IgG_1, k _antibody Isotype), PE conjugated mouse IgG1,_k _Isotype control (MOPC-21 21 clone and mouse IgG_1, k _antibody isotype), FITC conjugated Mouse IgG _2a,k _(G155-178 clone and mouse IgG _2a,k _antibody isotype and 7AAD were purchased from BD Pharmingen. CD14 microbead conjugated antibodies and columns were purchased from Miltenyi Biotech.

### Laboratory Methods

#### Generation of immature human Dendritic Cells in vitro

Immature DCs were generated from positively selected CD14^+ ^cells as described previously [6] with minor modifications. In brief, monocytes were purified by positive selection of CD14^+ ^cells from Peripheral Blood Mononuclear Cells (PBMCs) of apparently healthy Ethiopian male and female subjects by using a magnetic cell separator (VarioMacs, Miltenyi Biotech, Germany) according to the manufacturer's instruction. CD14^+ ^cells were then incubated in six-well tissue culture plates (at a concentration of approximately 2 × 10^6^/well in a volume of 3 ml) for 7 days at 37°C in an atmosphere of 5% CO_2 _in complete medium (RPMI 1640 with L-glutamine supplemented with 1% penicillin streptomycin and 10% FBS), 50 μM 2-mercaptoethanol, 50 ng/ml human recombinant GM-CSF and 50 ng/ml human recombinant IL-4. After 7 days of incubation, the non-adherent cells (Immature DCs) were harvested and placed in 24-well plates containing 2 ml complete medium without antibiotics. CD14^- ^cells were stored frozen in liquid nitrogen and used as autologus T cells to do mixed leukocyte reaction.

#### Mycobacteria preparation

H37Rv was grown in Middlebrook 7H10 agar (Difco Laboratories, Detroit, MI) at 37°C under a humidified 5% CO2 atmosphere for 2 weeks. Bacterial suspensions were prepared by dispersing colonies with glass beads in RPMI 1640. The tubes were vortexed and allowed to stand for 30 min to let larger particles settle. The upper supernatant was stored at -80°C until use. Colony-forming units were counted by the standard viable count technique in Middlebrook 7H10 agar plates.

#### Infection of DC with M. tuberculosis

Immature DC were plated in 24-well plates at 2 × 10^5 ^cells in 2 ml of complete media per well without antibiotics and then were infected with H37Rv at a multiplicity of infection (MOI) of 5 in the Biological Safety Level-3 (BSL-3) lab. After 6 hours of incubation at 37°C in 5% CO_2 _incubator, infected cells were washed three times with complete medium without antibiotics at low speed (600 rpm) to remove extracellular bacteria and were incubated in fresh medium for a further 2 days. Infected DC were incubated in 0.02% EDTA for 15 minutes at RT before harvesting to obtain the total cellular content of each well. This was followed by a single wash with complete media without antibiotics. The percentages of infected and necrotic cells were estimated in each experiment by staining aliquots of cells by Ziehl-Neelsen method for acid-fast bacteria and 7-amino-actinomycin D (7-AAD) respectively. In addition, infected DCs were evaluated after 48 hours for both cell surface phenotype and extent of autologous T cell stimulation using flow cytometry as described below.

#### Flow Cytometric Analysis of Surface Markers

All groups of DC (Immature DC alone, Immature DC infected with H37Rv *M. tuberculosis *strain, and stimulated with 1 μg/ml LPS for 48 hours) surface marker expression was assessed by flow cytometry (FACScan, BD) using direct double immunofluorescence labelling technique (CD40 FITC/CD80 PE, HLA DR FITC/CD54 PE, CD83 FITC/CD86 PE). In brief, 2 × 10^5 ^cells were aliquoted into tubes and washed once with FACS medium (PBS, 1% FBS and 0.1% Sodium azide) at 400 g at 4°C for 10 minutes and incubated with 15 μl fluorochrome conjugated monoclonal antibodies of each surface antigen for 30 minutes at 4°C. Then, cells were washed an additional three times with FACS medium. Dead cells were eliminated from the analysis by staining with 7-amino-actinomycin D (7AAD). The values were reported as Mean Fluorescence Intensity (MFI) of the total events counted (n = 10,000). DCs necrosis was assessed by 7-AAD Staining Assay. In all experiments evaluating DC phenotypic maturation, dead cells (7-AAD bright) were excluded from analysis.

### Cytokine assay

At the end of the incubation period, culture supernatants of DC were collected, filtered with 0.2 μm and stored at -80°C. The levels of TNF α, IL-12 and IL-10 were determined by enzyme-linked immunosorbent assay (ELISA) using recombinant cytokines for generating standard curves. Anti-human TNF α, IL-12 and IL-10 mAb, biotin mouse anti-human TNF α IL 12 and IL-10 mAb and standard were purchased from Pharmingen.

#### Mixed Leukocyte Reaction

The ability of *M. tuberculosis *infected DC to stimulate T cells was assessed using a mixed DC-autologous-T-cell reaction. Infected DCs were used to stimulate autologous CD 14^- ^PBMC (purified by magnetic beads following CD 14^+ ^positive selection). A total of 2 × 10^5 ^CFSE stained CD 14^- ^PBMC (responder cells) were added to each well in 100 μl complete media on U bottom, 96 well plates (BD, Pharmingen). DCs (stimulator cells) were harvested following stimulation with medium alone/LPS/*M. tuberculosis*, washed twice, resuspended in complete medium and then added to the responder cells at a ratio of 1:10 and 1:100. After 5 days culture in 5% CO_2 _at 37°C, cells were harvested and CD3 positive cells were analysed for their CFSE staining intensity.

The number of cells that had proliferated was determined by gating on the lineage positive CFSE^dim ^subset. The cell division index (CDI) was calculated based on a fixed number (usually 5,000) of CFSE^bright ^CD3^+ ^cells, with the following formula:

A CDI of greater than 2 was considered to represent a positive response. A CDI of 2.0 means that there are twice as many divided cells with antigen than without [7].

### Ethical Consideration

The project obtained ethical clearance from the Medical Faculty Research and Publication committee of the Addis Ababa University, AHRI/ALERT Ethical Review Committee and the National Ethical Review Committee before commencement of the study.

### Data Analysis

The mean fluorescence intensity data were analysed using a non-parametric Wilcoxon signed rank test, and results were considered significant if *p *≤ 0.05 and for the proliferation test CDI values greater than 2 was taken as a positive result [7]

## Results

### Viability of Dendritic Cells Following Infection with H37Rv

Immature DCs were infected with H37Rv at different multiplicity of infection (MOI). Twelve experiments from different subjects were done to determine the optimum MOI to be used in subsequent experiments. The percentage of the cells infected at the various MOIs at the time of collection varied from 26 to 89% (data not shown). Forty-eight hours after infection, cells were collected and viability was determined by staining with 7-AAD. Infected cells were stained with Zihel-Nielsen for the presence of intracellular acid-fast bacteria. At the lowest MOI of 3, only 31.6 ± 2.6% of the cells stained positive for intracellular bacteria and 5.6% were positive for 7-AAD. At MOI of 5, the percentage of cells staining positive for intracellular bacteria increased to 60 ± 1.3% and the percentage of cells positive for 7-AAD was 11.1 ± 4.5%, whereas at an MOI of 10, 84.4 ± 4.5% of cells stained positive for intracellular bacteria. At the highest MOI of 10, although the percentage of cells positive for intracellular bacteria was higher, a significant proportion (18.2%) of the DC were not viable. The mean percentage of viable immature DC was 97.7% (96.8-98.6%) in the case of uninfected cells. On the basis of these data, a MOI of 5 was chosen as optimal for further experiments.

### Maturation of Dendritic Cells

Functional activities of DC were determined by surface expression of adhesion and co-stimulatory molecules. To investigate the effect of *M. tuberculosis *H37Rv strain infection on the DC phenotype, the expression of the intracellular adhesion molecule (ICAM 1 and CD54), co-stimulatory surface molecules (B7-1 (CD80), B7-2 (CD86) and CD40) and MHC II by immature, H37Rv infected and LPS stimulated DC were assessed using 18 experiments from different subjects by flow cytometry. The cells were infected with H37Rv at a multiplicity of infection of 5 or stimulated with LPS at a concentration of 1 μg/ml or immature DC treated with media alone and analyzed 48 hr post treatment for the expression of the above-mentioned surface molecules. Significant (p < 0.05) up regulation in cell surface expression of CD40, CD80, CD54 and MHC class II and CD86 and CD83 (Table 1) were observed both in LPS stimulated and H37Rv infected in comparison to negative control DC with media alone without any stimulation/infection.

**Table 1:** Expression of various phenotypic markers on dendritic cells before and after infection

Phenotypic markers	Control (Uninfected)	LPS stimulated	H37Rv infected
**CD 40**	40.7 ± 16.4	153.3* ± 15.8	82.8* ± 12.8
**CD 80**	39.2 ± 6.7	109.5* ± 8.3	92.4* ± 6.9
**HLA DR**	153.4 ± 18.6	639.9* ± 12.7	480* ± 12.4
**CD 54**	102.3 ± 16.3	292.2* ± 13.4	218.7* ± 11.4
**CD 83**	3.2 ± 1.1	11.4* ± 2.4	8.1* ± 1.5
**CD 86**	22.4 ± 3.2	86.2* ± 6.2	78.4* ± 5.7

Expression of each marker is presented as percentage positive cells. The values represent mean ± SEM of at least 17 independent experiments for each condition. The statistical significance is shown as compared to uninfected; *P < 0.05

### Cytokine ELISA

**Figure 1:**
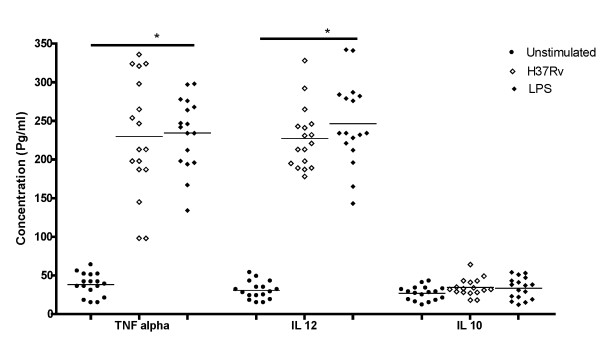
**Cytokine Secretion by MoDCs infected with M. tuberculosis and stimulated with LPS**. The production of TNF α and IL-12 was found to be significantly higher (P < 0.05) in all H37Rv infected and LPS stimulated MoDC compared to control whereas the production of IL--10 was not statistically significant (P > 0.05).

The production of TNF α and IL 12 was found to be significantly higher in all infected MoDC compared to control (Figure 1). IL10 was also measured in the MoDC culture supernatants but their levels were not statistically significant compared to unstimulated groups (P > 0.05).

### Mixed Leukocyte Reaction

The ability of *M. tuberculosis *infected DC to stimulate autologous T cells was assessed using mixed DC-autologous T cell reaction. The T cell proliferation was quantified by CFSE dilution techniques described in the methodology part. The results were expressed as the mean Cell Division Index (CDI) from each experiment.

**Figure 2:**
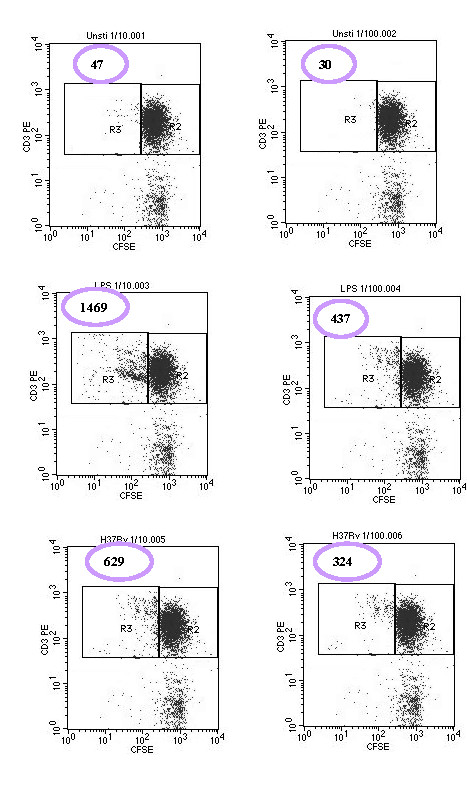
**Autologous T cell proliferation at 1:10 and 1:100 of DC: T cell ratio by CFSE dilution technique**. The cells were stained with antiCD3 PE and analysed with flow cytometry. In all cases 5000 CD3^+ ^CFSE bright events collected (Right Hand Box (R2)); the Number of CD3^+ ^CFSE ^dim ^events (Left Hand Box (R3)) was then determined. The cell division index (CDI) was calculated as described in the methodology.

The DC stimulated with LPS and infected with H37Rv augments T cells proliferation both at 1:10 (Mean CDI = 34.2 and 15) and 1:100 (Mean CDI = 15.6 and 10.6) DC: T cell ratio respectively (Figure 2).

## Discussion

The DC activation process that results in a mature phenotype appears to be a crucial step in generating a specific immune response. We used the virulent *M. tuberculosis *H37Rv strain to infect DC. The percentage of infected DC was proportional to the MOI. In this study, we used a MOI of 5, which resulted in 60 ± 1.3% level of infection. Higher MOI of 12.5 caused a large amount of necrosis [8]. In our study we have found 5.6%, 11.1% and 18.2% of cell death at a MOI of 3, 5 and 10 respectively, which clearly shows at higher MOI the more cell death will be.

This study demonstrates that the exposure of monocyte-derived immature DC to H37Rv *M. tuberculosis *strain leads to activation or maturation of DC. Significant increases in cell surface expression of accessory molecules, CD40, CD80, CD86 and CD83, CD54), and MHC class II maturation marker (p < 0.05) were observed. The increase in the expression of CD40, CD80 and CD86 may result in an increased capacity of DC to trigger proliferative responses and IFN-γ production by T cells against *M. tuberculosis *infection. In addition, CD40-CD40L interaction is the most potent stimulus in up regulating the expression of CD54, CD80, and CD86 molecules on DC [9].

The increment of expression of CD54 would help to increase the strength of adhesion between T cells and DC, thus allowing the TCR to be engaged by antigen to transduce the necessary signals, since it is known that the affinity of TCR for peptide MHC complex is quite low and the off rate of this interaction is also rapid.

Although the precise function of CD83 is still unknown, several pathogens such as HSV-1 [10] and Measles virus [11] have been shown to interfere with CD83-expression in infected DC, which then consequently also interfered with DC-mediated T-cell stimulation. Furthermore, in experiments where the CD83 mRNA transport from the nucleus into the cytoplasm was specifically inhibited, and thus CD83 expression was blocked, the T-cell stimulatory capacity of these DC was also inhibited [12]. All these reports clearly suggested an important role for CD83 during the induction of immune responses. The up regulation of HLA DR molecules on the surface of DC may also ensures that T cells will have the chance to recognize and respond to MHC associated peptides. In general, up regulation of these cell surface markers that are important in antigen presentation and T cell stimulation indicates that the dendritic cells are maturing and preparing for the presentation of antigen to T lymphocytes.

Other workers also reported that DC readily internalized *M. tuberculosis *bacilli and subsequently displayed phenotypic changes including up regulation of various cell surface molecules important in initiating immune responses and downregulation of phagocytic activity, as well as producing inflammatory cytokines [12].

Other workers have also shown that pulmonary DC traffic from the lungs to the draining lymph nodes to present inhaled antigens to T cells after bacterial infection [13]. In response to *M. tuberculosis *infection, DC shift to an antigen presenting phenotype and can stimulate T cells from the spleens and lungs of infected mice *in vitro *[14]. *M. tuberculosis *infected DCs have been observed *in vivo *in infected mice [15].

In contrast to our findings, other workers have reported that *M. tuberculosis *targets DC-specific C-type lectin intercellular adhesion molecule-3-grabbing nonintegrin (DC-SIGN) both to infect DC and to down-regulate maturation of human monocyte-derived DCs. *M. tuberculosis *induced minimal up regulation of the chemokine receptor CCR7, the co-stimulatory molecules CD40, CD80, and CD86 and the antigen presenting molecules MHC class I and MHC class II [16].

The discrepancy between the previous reports and this study may arise from two points: multiplicity of infection and the positive control that was used. The MOI that they used was 3 with an infection of approximately 25%. This indicates a low proportion of infection and with a possibility of very few bacilli per cell, which consequently could lead to a false negative or low maturation of DC. The other point is that they compared the state of maturation or MFI of *M. tuberculosis *infected DC with DC stimulated with a maturation cocktail and this optimal positive control would give a higher MFI as compared to *M. tuberculosis*. However, we used a MOI of 5 that would give relatively high number of bacilli per cell and we used a bacterial cell wall component (LPS) as a positive control, which would possibly give us a picture more comparable to natural pathogens.

We also examined the ability of *M. tuberculosis *infected DC to induce antigen specific T cell proliferation. Maturation does not necessarily mean that the cell is functionally capable of presenting antigen to T cells. The ability to measure autologous T cell proliferation in response to antigen was assessed by the CFSE dilution technique. Both at 1:10 and 1:100 DC: T cell ratio, infected DC induced positive T cell proliferation (CDI > 2), although T cell proliferation was stronger at a 1:10 rather than 1:100 DC: T cell ratio, which could in fact be due to the higher number of DCs. The ability of H37Rv infected DCs to induce T cell proliferation was slightly lower than LPS and this may be due to the higher concentration of LPS that we used to stimulate dendritic cells.

## Conclusion

In this study, we have clearly demonstrated that *M. tuberculosis *infection resulted in a phenotype change and production of proinflammatory cytokines that lead to maturation and possibly to migration and effective antigen processing and presentation with a polarization of the immune response towards protective Th-1 type. We have also demonstrated that *M. tuberculosis*-infected DCs are capable of inducing T cell proliferation. In general, DCs could be the basis for the initiation of the immune response leading to protection in the majority of *M. tuberculosis*-infected individuals. However, this aspect requires further investigation.

## Competing interests

The authors declare that they have no competing interests.

## Authors' contributions

AM carried out all the laboratory work, analyzing data and preparing the manuscript. SP contributed in conception, designing the study and correcting the manuscript. GM and MS contributed in laboratory work. AH contributed in guiding laboratory work and correcting the manuscript. All authors read and approved the final manuscript.
